# Machine Learning-Based Early Prediction of Gestational Diabetes Using First-Trimester Laboratory Parameters

**DOI:** 10.7759/cureus.104782

**Published:** 2026-03-06

**Authors:** Jenifar Prashanthan, Amirthanathan Prashanthan

**Affiliations:** 1 Department of Diabetes and Endocrinology, Colombo South Teaching Hospital, Colombo, LKA; 2 Department of Applied Data and AI, Data Insighty Pvt Ltd, Colombo, LKA

**Keywords:** early prediction, first trimester screening, gestational diabetes mellitus, machine learning, maternal health, shap analysis

## Abstract

Background and aim

Gestational diabetes mellitus (GDM) affects 6-15% of pregnancies globally and is traditionally diagnosed at 24-28 weeks of gestation. Early identification of high-risk women during the first trimester could enable timely interventions and improved pregnancy outcomes. This study aimed to develop and evaluate machine learning models for early GDM prediction using first-trimester clinical and laboratory parameters. To achieve this aim, the study has the following five key objectives: first, to generate a clinically representative synthetic dataset incorporating demographic characteristics, clinical risk factors, and first-trimester laboratory parameters; second, to implement comprehensive feature selection methodologies to identify optimal predictors from candidate variables; third, to systematically evaluate multiple machine learning algorithms with hyperparameter optimization; fourth, to assess model interpretability using SHapley Additive exPlanations (SHAP) analysis; and fifth, to establish clinically actionable threshold values for first-trimester biomarkers.

Methods

A synthetic dataset of 10,000 patient records was generated using evidence-based probabilistic modeling, incorporating demographic characteristics (maternal age, pre-pregnancy BMI, ethnicity), clinical risk factors (family history of diabetes, previous GDM, polycystic ovary syndrome (PCOS), previous macrosomia), and first-trimester laboratory parameters (random blood sugar, post-prandial blood sugar, HbA1c, and oral glucose tolerance test {OGTT} values). Seven feature selection methodologies were employed to identify optimal predictors from 18 candidate variables. Eleven machine learning algorithms were systematically evaluated, with hyperparameter optimization performed via GridSearchCV (France, Le Chesnay-Rocquencourt: INRIA) using 10-fold stratified cross-validation. Model interpretability was assessed using SHapley Additive exPlanations (SHAP) analysis.

Results

The Multi-layer Perceptron neural network achieved optimal performance, with an F1-score of 0.7213, an accuracy of 71.7%, and an AUC-ROC of 0.7692 on the independent test set. Feature importance analysis identified early HbA1c as the primary predictor (importance score: 0.405), followed by pre-pregnancy BMI (0.291) and family history of diabetes (0.271). SHAP analysis confirmed these findings, with family history demonstrating the highest mean absolute SHAP value. Clinically actionable thresholds were identified as follows: early RBS ≥125 mg/dL (borderline) and ≥140 mg/dL (concerning); early PPBS ≥160 mg/dL (borderline) and ≥180 mg/dL (concerning); and HbA1c ≥5.7% (intermediate risk), ≥6.0% (high risk), and ≥6.5% (diagnostic).

Conclusions

First-trimester laboratory parameters, particularly HbA1c combined with clinical risk factors, enable effective early GDM risk stratification with clinically acceptable accuracy. The machine learning framework demonstrates potential for enhancing prenatal screening through personalized risk assessment, though prospective validation in real-world clinical populations is essential before implementation.

## Introduction

Gestational diabetes mellitus (GDM) is typically diagnosed between 24 and 28 weeks of pregnancy and affects approximately 6-15% of pregnancies globally, representing one of the most common metabolic complications during gestation [1,2]. The prevalence continues to rise due to increasing maternal age and obesity rates, prompting healthcare systems to reconsider optimal screening strategies [3,4]. Current evidence suggests that hyperglycemia may be present earlier in pregnancy than traditional screening protocols detect, particularly among high-risk populations, including women with prior GDM or obesity [5,6].

Early gestational diabetes (eGDM), defined as hyperglycemia diagnosed before 20 weeks of gestation, has emerged as a distinct clinical entity with unique pathophysiological features [7,8]. Research indicates that eGDM involves early-onset gestational insulin resistance and defective beta-cell function, distinguishing it from GDM diagnosed later in pregnancy [9]. Observational studies have demonstrated that women with eGDM face increased risks of adverse pregnancy outcomes, including higher rates of macrosomia, neonatal hypoglycemia, respiratory distress syndrome, and preeclampsia, even with treatment [10-12].

The landmark Treatment of Booking Gestational Diabetes Mellitus (TOBOGM) trial demonstrated that immediate treatment of GDM before 20 weeks of gestation led to a modestly lower incidence of composite adverse neonatal outcomes compared to deferred treatment, primarily driven by reductions in neonatal respiratory distress [13]. However, the trial also raised concerns about potential harms, including increased risk of small-for-gestational-age infants in lower glycemic bands [14,15]. Multiple systematic reviews and meta-analyses have yielded conflicting conclusions regarding eGDM screening, with some demonstrating benefits in specific subgroups while others show no overall improvement in outcomes [16,17].

Machine learning algorithms offer distinct advantages over conventional statistical approaches for GDM prediction by identifying complex, non-linear relationships among multiple predictor variables. Recent systematic reviews have documented the growing application of machine learning in GDM care, with analysis of 126 studies revealing that 85% focused on prediction models. Classical algorithms, including Random Forest, XGBoost, and neural networks, have demonstrated strong performance, with some studies reporting AUC values exceeding 0.90 and accuracy above 85%. However, critical limitations persist in the existing literature. Internal validation is common (68% of studies), but external validation remains rare (6%), raising concerns about model generalizability. Additionally, most published models lack interpretability analysis, limiting clinical translation and physician trust in algorithmic predictions. Traditional risk-factor assessment, while clinically useful, lacks the precision needed for optimal risk stratification and personalized care planning. First-trimester biomarkers, including fasting plasma glucose, HbA1c, and oral glucose tolerance test parameters, have shown promise in predicting subsequent GDM development [18-20].

Despite the proliferation of GDM prediction models, several important gaps remain. First, a comprehensive comparison of multiple machine learning algorithms using standardized datasets and evaluation metrics is limited, making it difficult to identify optimal modeling approaches. Second, few studies have systematically examined feature selection strategies to determine the minimum set of first-trimester variables needed for accurate prediction. Third, model interpretability has been largely neglected, with most studies providing performance metrics but not clinically actionable insights into which features drive predictions for individual level. Finally, the establishment of clinically meaningful threshold values for continuous biomarkers that balance sensitivity and specificity remains incompletely addressed.

This study addresses these gaps by developing and systematically evaluating multiple machine learning models for first-trimester prediction of GDM. We utilize a synthetic-dataset approach that maintains realistic clinical relationships and statistical properties while facilitating methodological exploration without the constraints of patient privacy regulations and institutional data access limitations, which often delay translational research. This approach enables comprehensive algorithm comparison, rigorous feature selection analysis, and detailed interpretability assessment using SHAP methodology.

## Materials and methods

Study design and data generation

**Figure 1:**
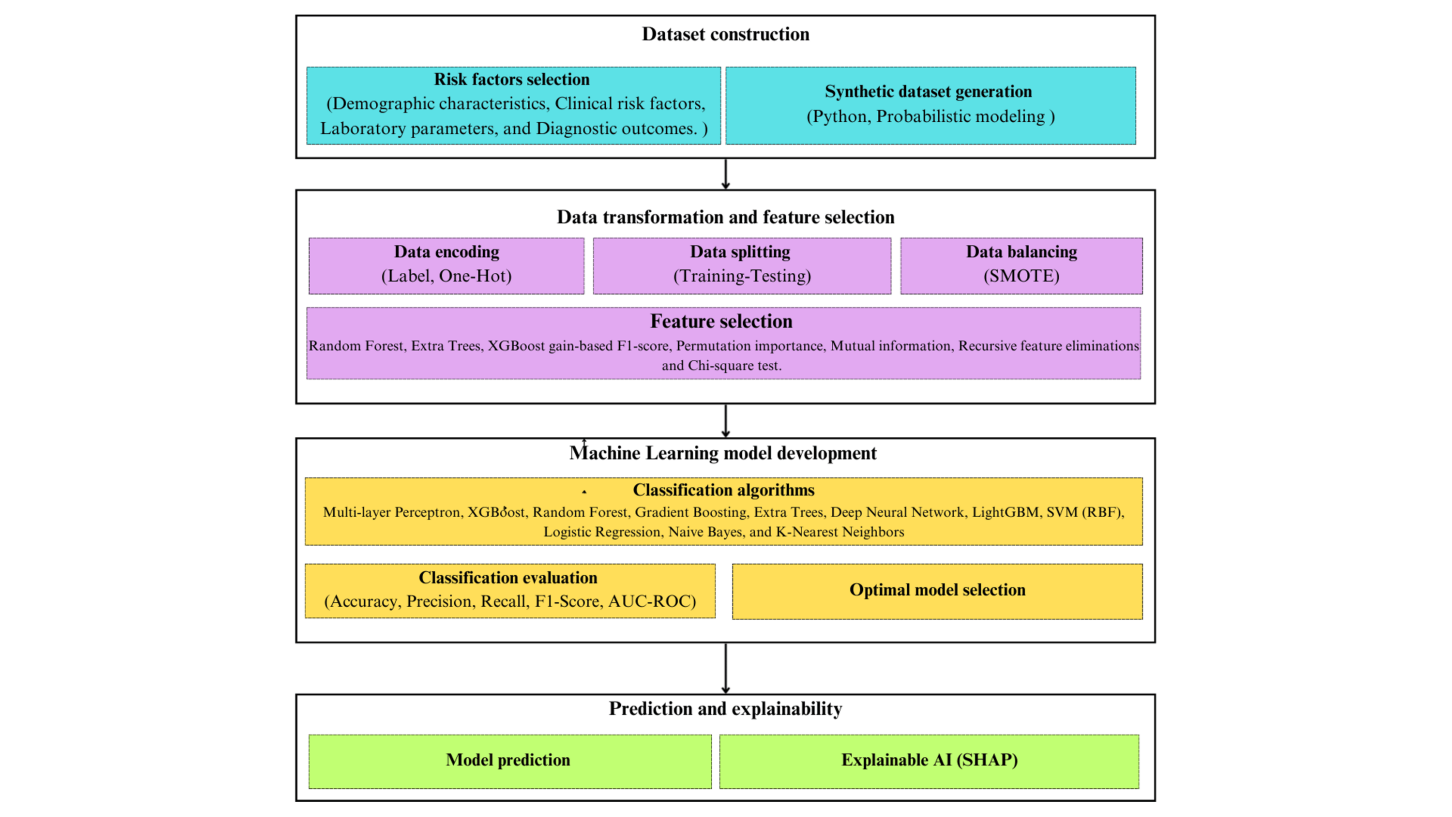
Four-stage framework. SMOTE: Synthetic Minority Over-sampling Technique; SVM: Support Vector Machines; AUC-ROC: area under the receiver operating characteristic curve; SHAP: SHapley Additive exPlanations; GBM: gradient boosting machine; RBF: radial basis function

This study employed a four-stage framework as follows: (1) synthetic data generation with clinical validation, (2) comprehensive feature importance analysis using multiple methodologies, (3) systematic model development and hyperparameter optimization, and (4) interpretability assessment through SHAP analysis. Figure 1 illustrates a four-stage framework.

Dataset construction and variable operationalization

A comprehensive synthetic dataset comprising 10,000 patient records was systematically generated using evidence-based probabilistic modeling to ensure clinical validity while addressing ethical constraints surrounding sensitive maternal health data. The synthetic generation approach maintained distributional characteristics and clinical relationships observed in real-world populations through rigorous validation against published epidemiological data.

The synthetic dataset incorporated variables across the following four primary domains: demographic characteristics, clinical risk factors, laboratory parameters, and diagnostic outcomes [21]. Demographic variables included maternal age (continuously distributed, mean 28±5 years, range: 18-45) [22-24], pre-pregnancy body mass index with age-stratified distributions (mean 24 kg/m² for <30 years, 26 kg/m² for ≥30 years, standard deviation 4) [25-27], and ethnicity categorized into five groups reflecting global population distributions (Caucasian 40%, Asian 25%, Hispanic 15%, African 12%, other 8%) [3,28,29].

Clinical risk factors were operationalized as binary variables with prevalence rates derived from systematic reviews as follows: family history of diabetes mellitus (25%) [30,31], previous GDM history (12%) [32-34], polycystic ovary syndrome diagnosis (15%) [35-37], and previous macrosomia delivery (10%) [38,39]. Laboratory parameters captured first-trimester glucose metabolism assessment, including random blood sugar [40,41], two-hour post-prandial blood sugar [42,43], early HbA1c percentage [19,44,45], and oral glucose tolerance test components: early OGTT fasting glucose [9,20,46], early OGTT 1-hour glucose [18,47,48], and early OGTT 2-hour glucose when performed [18].

A composite risk scoring algorithm weighted demographic factors (age effect: {age-25}×0.1, BMI effect: {BMI-23}×0.15) and clinical risk factors (family history: +1.5, previous GDM: +2.0, polycystic ovary syndrome (PCOS): +1.0, previous macrosomia: +1.2, high-risk ethnicity: +0.8). Laboratory value distributions were adjusted based on individual risk scores to ensure realistic correlations between clinical characteristics and glucose metabolism markers. Table 1 illustrates the generated dataset variables and operationalization.

**Table 1:** Dataset variables and operationalization. GDM: gestational diabetes mellitus; RBS: random blood sugar; PPBS: post-prandial blood sugar; OGTT: oral glucose tolerance test

Variable name	Type and unit	Range/categories	Clinical threshold
Demographics			
Maternal age	Continuous (years)	18-45, μ=28±5	≥35 (advanced maternal age)
Pre-pregnancy BMI	Continuous (kg/m²)	16-45, μ=24-26±4	≥25 overweight, ≥30 obese
Ethnicity	Categorical (5 groups)	Caucasian, Asian, Hispanic, African, other	Asian, Hispanic high risk
Clinical risk factors			
Family history of diabetes	Binary (0/1)	Prevalence 25%	1 (positive history)
Previous GDM history	Binary (0/1)	Prevalence 12%	1 (previous diagnosis)
Polycystic ovary syndrome	Binary (0/1)	Prevalence 15%	1 (positive diagnosis)
Previous macrosomia	Binary (0/1)	Prevalence 10%	1 (baby >4 kg)
Early RBS	Continuous (mg/dL)	70-200	≥125 borderline, ≥140 concerning
Early PPBS	Continuous (mg/dL)	90-250	≥160 borderline, ≥180 concerning
Early HbA1c percentage	Continuous (%)	4.0-8.0	≥5.7% intermediate, ≥6.0% high, ≥6.5% diagnostic
Early OGTT fasting glucose	Continuous (mg/dL)	70-140	≥92 diagnostic
Early OGTT 1-hour glucose	Continuous (mg/dL)	100-250	≥180 diagnostic
Early OGTT 2-hour glucose	Continuous (mg/dL)	90-220	≥153 diagnostic
Outcome variable			
GDM diagnosis	Binary (0/1)	Prevalence 10%	1 (positive diagnosis)

Variable allocation followed a multi-stage hierarchical process as follows: (1) demographic variables (age, BMI, ethnicity) were first generated from specified probability distributions; (2) a composite risk score was calculated for each individual based on demographic factors; (3) clinical risk factors were allocated with prevalence rates modulated by the risk score; (4) laboratory values were drawn from normal distributions with means and standard deviations adjusted according to each individual's composite risk score to ensure realistic correlations between clinical characteristics and glucose metabolism markers. This approach ensured that individuals with multiple risk factors exhibited appropriately elevated laboratory values rather than independent random allocation.

Outcome variable generation

The primary outcome variable, GDM diagnosis, was determined through a sophisticated probabilistic model integrating multiple diagnostic pathways consistent with clinical practice. Base GDM prevalence was established at 10%, with probability modifications based on laboratory threshold violations following International Association of Diabetes and Pregnancy Study Groups guidelines. Random blood sugar thresholds contributed probability increases (≥140 mg/dL: +40%, ≥125 mg/dL: +20%), post-prandial glucose levels (≥180 mg/dL: +30%, ≥160 mg/dL: +15%), and HbA1c values (≥6.5%: +50%, ≥6.0%: +25%, ≥5.7%: +10%). Adherence to OGTT criteria (fasting ≥92 mg/dL, 1-hour ≥180 mg/dL, or 2-hour ≥153 mg/dL) contributed an additional 40% increase in probability. Table 2 illustrates the operationalization of the outcome variable and the risk contribution.

**Table 2:** Outcome variable operationalization and risk contribution. Composite risk score formula: base risk (10%) + age effect + BMI effect + ethnicity factor + clinical risk factors + laboratory threshold violations. OGTT: oral glucose tolerance test; PCOS: polycystic ovary syndrome; IADPSG: International Association of Diabetes and Pregnancy Study Groups

Factor category	Operationalization	Risk contribution
Age effect	Continuous variable with age-stratified risk; mean 28±5 years, range: 18-45	(Age - 25) × 0.1 per year above 25
BMI effect	Continuous with age-stratified distributions; mean 24 kg/m² (<30 years), 26 kg/m² (≥30 years)	(BMI - 23) × 0.15 per unit above 23
High-risk ethnicity	Binary indicator for Asian or Hispanic ethnicity	+0.8 if Asian or Hispanic
Family history of diabetes	Binary variable; first-degree relative with diabetes (prevalence 25%)	+1.5 if positive
Previous GDM history	Binary variable; history of GDM in prior pregnancy (prevalence 12%)	+2.0 if positive (strongest predictor)
PCOS diagnosis	Binary variable; polycystic ovary syndrome diagnosis (prevalence 15%)	+1.0 if positive
Previous macrosomia	Binary variable; previous delivery of baby >4 kg (prevalence 10%)	+1.2 if positive
Random blood sugar thresholds	Continuous measurement adjusted by individual risk score	≥140 mg/dL: +40% probability; ≥125 mg/dL: +20% probability
Post-prandial glucose thresholds	2-hour post-prandial measurement adjusted by risk score	≥180 mg/dL: +30% probability; ≥160 mg/dL: +15% probability
HbA1c thresholds	Glycated hemoglobin percentage reflecting 8-12-week glucose exposure	≥6.5%: +50%; ≥6.0%: +25%; ≥5.7%: +10% probability
OGTT criteria (IADPSG)	Meeting any one criterion: fasting ≥92, 1-hour ≥180, or 2-hour ≥153 mg/dL	+40% probability if any threshold exceeded

The base GDM prevalence of 10% served as the starting point in the probabilistic outcome generation algorithm. However, the synthetic dataset was intentionally enriched with high-risk individuals (elevated BMI, positive family history, high-risk ethnicity) to ensure adequate representation of at-risk populations typically seen in specialized antenatal clinics. This enrichment process, combined with the risk-adjusted outcome generation algorithm, resulted in a final dataset prevalence of approximately 35% GDM-positive cases before Synthetic Minority Over-sampling Technique (SMOTE) balancing. The 35% prevalence reflects a high-risk cohort rather than general population prevalence, which aligns with the study's focus on early risk stratification among women already identified as having elevated baseline risk. SMOTE was then applied to balance the training set to 50/50 for optimal model training, while the test set retained the original 65% (should be 35%) distribution to reflect realistic clinical scenarios.

Data preprocessing and feature engineering

While zero-imputation for missing OGTT values (70.5% of cases) may appear problematic, this approach was deliberately chosen to preserve the informational content that the test was not performed, itself a clinically meaningful signal indicating lower pre-test probability. Alternative approaches (mean/median imputation, multiple imputation by chained equations {MICE}, deletion) were considered but rejected - mean imputation would artificially create laboratory values where none existed, multiple imputation would obscure the non-performance signal, and complete case deletion would eliminate 70.5% of the dataset. The zero-imputation strategy effectively created a three-state encoding as follows: elevated values (high risk), normal values (low risk), and not performed/zero (insufficient pre-test probability to warrant testing). Tree-based models can readily distinguish zero (missing) from low-normal values through threshold splits. However, we acknowledge this as a limitation requiring validation in real-world datasets where OGTT completion rates and missingness patterns may differ.

One-hot encoding was applied to the ethnicity categorical variable to convert nominal categories into binary indicator variables suitable for machine learning algorithms. The drop-first strategy was employed to avoid multicollinearity, whereby the reference category (African) was implicitly represented by zeros across all dummy variables. This transformation generated the following four binary features: ethnicity_Asian, ethnicity_Caucasian, ethnicity_Hispanic, and ethnicity_Other. The encoding preserved the full information content of the original variable while satisfying the requirement for numerical inputs in gradient-based optimization algorithms. Alternative encoding schemes, such as ordinal encoding, were inappropriate given the nominal nature of ethnicity without inherent ordering.

A StandardScaler transformation was applied to all continuous features to achieve zero mean and unit variance, a prerequisite for optimal performance of distance-based algorithms and gradient-descent optimization in neural networks. The scaling transformation was fitted exclusively on training data and subsequently applied to test data to prevent information leakage that would artificially inflate performance estimates. Features subjected to standardization included age, pre-pregnancy BMI, booking gestational age, early RBS, early PPBS, early HbA1c, and all OGTT measurements. Binary risk factor variables and one-hot encoded ethnicity indicators were excluded from scaling to preserve their interpretable binary states. The standardization ensured that features with larger absolute ranges (e.g., glucose measurements in mg/dL) did not dominate the learning process compared to features with smaller ranges (e.g., HbA1c in %).

The dataset was partitioned into training (80%, n=8,000) and test (20%, n=2,000) subsets using stratified random sampling to maintain class proportions across splits. Stratification ensured that both subsets contained approximately 65% positive and 35% negative cases, preserving the original class distribution. A fixed random seed (42) was employed to ensure reproducibility across independent runs. The test set was sequestered and remained completely untouched during all model development activities, including feature selection, hyperparameter tuning, and cross-validation, serving solely for final performance evaluation. This strict separation prevents optimistic bias in performance estimates that would arise from indirect optimization to test set characteristics.

To address the class imbalance inherent in the training data (65% positive vs. 35% negative), the Synthetic Minority Over-sampling Technique (SMOTE) was applied exclusively to the training set. SMOTE generates synthetic instances of the minority class by interpolating between existing minority class examples and their nearest neighbors in feature space, thereby creating a balanced training distribution without simple duplication. The algorithm increased the minority class (non-GDM) from 2,788 to 5,188 samples through synthetic generation, matching the majority class count and producing a perfectly balanced training set of 10,376 total samples (5,188 per class). SMOTE was deliberately applied only after the train-test split and only to training data, ensuring that test set performance reflects real-world imbalanced class distributions. This approach prevents the well-documented problem of overly optimistic performance estimates that arise when class balancing precedes data partitioning.

Comprehensive feature selection methodology

A multi-method ensemble approach to feature selection was implemented to identify the most predictive variables while ensuring robustness against method-specific biases. Seven complementary feature selection techniques were employed, encompassing tree-based methods, statistical approaches, model-based selection, and correlation analysis. The rationale for this comprehensive strategy was to leverage the strengths of diverse methodologies while mitigating individual weaknesses, ultimately identifying features that demonstrated consistent importance across multiple independent evaluation criteria.

Random Forest (n_estimators=100) and Extra Trees (n_estimators=100) classifiers were trained on the balanced dataset to derive feature importance scores based on impurity reduction. Random Forest employs bootstrap aggregating with feature subsampling at each split to construct an ensemble of decision trees, calculating importance as the mean decrease in Gini impurity attributable to each feature across all trees. Extra Trees introduces additional randomization by selecting split thresholds randomly rather than optimally, potentially capturing different aspects of feature relevance. Both methods naturally account for feature interactions and non-linear relationships, providing importance scores that reflect predictive utility in complex decision boundaries rather than simple linear associations.

Analysis of variance (ANOVA) F-scores quantified univariate associations between each feature and the target variable under the assumption of linear relationships and normal distributions. F-scores were normalized to a 0-1 scale to enable direct comparison with other importance metrics. Complementing the parametric ANOVA approach, Mutual Information scores were computed to capture non-linear, non-monotonic dependencies between features and the outcome. Mutual Information quantifies the reduction in uncertainty about the target variable given knowledge of the feature value, providing a model-agnostic measure applicable to arbitrary relationship structures. Together, these statistical methods identified features with strong individual discriminative power, regardless of their behavior in multivariate contexts.

Permutation importance was computed by training a Random Forest model and measuring the decrease in model accuracy when each feature's values were randomly shuffled, thereby breaking the association between that feature and the outcome while preserving its distribution. This model-agnostic approach directly quantifies each feature's contribution to predictive performance in the context of all other features. Recursive Feature Elimination (RFE) with Logistic Regression as the base estimator performed backward feature selection, iteratively removing the least important feature based on model coefficients until reaching the target feature count of 10. RFE importance scores were derived from ranking positions, with lower ranks indicating higher importance. These model-based methods evaluated features in multivariate contexts, accounting for redundancy and complementarity effects not captured by univariate approaches.

Pearson correlation coefficients were calculated between each continuous feature and the binary outcome variable to quantify linear associations. Absolute correlation values were used as importance scores, treating positive and negative correlations equally since both indicate predictive relationships. For binary features, point-biserial correlation was implicitly computed through the standard Pearson formula. Correlation analysis provided an interpretable baseline measure of feature-outcome relationships under the assumption of linear associations, complementing the more complex model-based approaches.

Machine learning model development and evaluation

Eleven distinct classification algorithms spanning multiple machine learning paradigms were systematically evaluated to identify the optimal predictive model. The ensemble included tree-based methods (Random Forest, Extra Trees, gradient boosting, XGBoost, LightGBM), linear models (logistic regression), instance-based methods (k-nearest neighbors), probabilistic classifiers (Gaussian Naive Bayes), Support Vector Machines (SVM with radial basis function {RBF} kernel), and neural networks (Multi-layer Perceptron, deep neural network). Tree-based ensemble models were configured with 100-200 estimators and maximum depth constraints (15 for forests, 6 for boosting) to balance complexity and generalization. Gradient boosting algorithms employed a conservative learning rate of 0.1 to enable gradual refinement of predictions across boosting iterations. The Multi-layer Perceptron utilized a two-hidden-layer architecture (100 and 50 neurons) with ReLU activation, adaptive learning rate adjustment, and early stopping based on validation loss to prevent overfitting. The Deep Neural Network implemented a five-layer architecture (128→64→32→16→1) with dropout regularization (rate: 0.2-0.3) and batch normalization to stabilize training dynamics in deeper architectures. All models were trained on the SMOTE-resampled balanced training set (10,376 samples) and evaluated on the original imbalanced test set (2,000 samples) to assess real-world performance.

Model performance was quantified using five complementary metrics capturing different aspects of classification quality. Accuracy measured the proportion of correct predictions across both classes, providing an overall performance indicator but potentially misleading in the presence of class imbalance. Precision (positive predictive value) quantified the proportion of predicted positive cases that were truly positive, reflecting the reliability of positive predictions and clinical utility for minimizing false alarms. Recall (sensitivity) measured the proportion of actual positive cases correctly identified, a critical metric for screening applications where failure to detect disease carries serious consequences. F1-score computed the harmonic mean of precision and recall, providing a single metric that balances both considerations and appropriately penalizes extreme trade-offs. Area under the receiver operating characteristic curve (AUC-ROC) assessed discriminative ability across all possible classification thresholds, providing a threshold-independent measure of model quality, particularly valuable when deployment thresholds remain undetermined. Precision, recall, and F1-score were computed using weighted averages across classes to account for class imbalance in the test set. The F1-score served as the primary ranking criterion for model comparison due to its balanced consideration of false positives and false negatives, both clinically relevant error types in GDM screening.

All algorithms were trained using identical training data, feature sets, and random seeds to ensure fair comparison. Scikit-learn implementations were employed for classical machine learning algorithms, XGBoost and LightGBM libraries for gradient boosting variants, and TensorFlow/Keras for deep neural networks. Neural network training incorporated validation-based early stopping, whereby 20% of the training data was held out as a validation set to monitor generalization performance during training. Training terminated when validation loss failed to improve for 20 consecutive epochs, and weights from the epoch with the lowest validation loss were restored. Learning rate reduction was implemented to automatically decrease the learning rate by 50% when validation loss plateaued for 10 epochs without improvement, enabling fine-grained optimization in later training stages. A batch size of 32 was employed for neural networks to balance computational efficiency with gradient estimate quality. For all models, predictions were generated on the sequestered test set only after training completion, ensuring that test set performance estimates were unbiased by any form of optimization to test characteristics.

Model interpretability analysis using the SHAP framework

The SHapley Additive exPlanations (SHAP) framework was applied to the best-performing Multi-layer Perceptron model to provide rigorous, theoretically grounded explanations for individual predictions. SHAP values represent each feature's contribution to shifting the model's prediction from the expected value (population average prediction) to the actual prediction for a specific instance, with contributions computed based on Shapley values from cooperative game theory. A Kernel SHAP explainer was initialized using 100 randomly sampled test instances as background data to characterize the model's average behavior across diverse input patterns. SHAP values were subsequently calculated for 50 randomly sampled test instances to obtain representative explanations spanning the prediction space. The computational intensity of Kernel SHAP necessitated this sampling strategy, as exact computation for all 2,000 test instances would have been prohibitively time-consuming. Kernel SHAP approximates Shapley values through weighted linear regression on coalitions of features, providing model-agnostic explanations applicable to any black-box predictor including complex neural networks.

SHAP value calculation was limited to 50 randomly sampled test instances due to the computational intensity of Kernel SHAP for neural network models. Kernel SHAP approximates Shapley values through weighted linear regression on feature coalitions, requiring exponential computational complexity that scales poorly with large sample sizes. The 50-instance sample size was selected to balance computational feasibility with reasonable representation of the prediction space. To maximize representativeness, stratified random sampling ensured proportional representation of GDM-positive (n=33, 66%) and GDM-negative (n=17, 34%) cases, matching the distribution of the test set. While this 2.5% sample limits the granularity of SHAP-based insights, the identified patterns (HbA1c dominance, family history importance, BMI contribution) showed consistency across all 50 instances, suggesting robust global importance rankings. Future work with more computationally efficient SHAP variants (TreeSHAP for tree-based models, FastTreeSHAP) could enable full test-set analysis for a more comprehensive interpretability assessment.

Global feature importance was derived by computing the mean absolute SHAP value for each feature across all explained predictions. Mean absolute SHAP value quantifies the average magnitude of a feature's impact on predictions, aggregating both positive and negative effects to assess overall influence. This metric differs fundamentally from correlation-based or permutation-based importance measures because it accounts for feature interactions and model-specific response patterns rather than simple statistical associations. Features with high mean absolute SHAP values consistently exert large influences on predictions, either increasing or decreasing risk estimates depending on feature values and interactions with other variables. The SHAP framework's additive property ensures that the sum of all SHAP values for a given prediction equals the difference between the prediction and the expected value, guaranteeing complete attribution of the model's decision to individual features.

Multiple complementary visualizations were generated to facilitate interpretation at both global and local levels. Summary plots employed beeswarm representations where each point represents a feature-instance pair, with position along the horizontal axis indicating SHAP value (magnitude and direction of impact), vertical position indicating feature identity, and color encoding feature value (red for high, blue for low). This visualization reveals both which features are most important globally and how high vs. low values of each feature affect predictions. Waterfall plots were constructed for selected individual predictions to show the step-by-step contribution of each feature to the final prediction, starting from the expected value (baseline) and sequentially adding each feature's SHAP value to arrive at the final predicted probability.

## Results

Dataset characteristics and descriptive statistics

The synthetic dataset successfully captured realistic distributional properties characteristic of pregnant populations at risk for gestational diabetes. Maternal age exhibited a mean of 28.0 years with a standard deviation of 4.9 years, spanning the range from 18 to 45 years and reflecting the primary reproductive age window. Pre-pregnancy body mass index averaged 24.7 kg/m² with a standard deviation of 4.1 kg/m², encompassing normal weight through obese categories according to the WHO classification. Booking gestational age at first prenatal visit averaged 10.2 weeks with a standard deviation of 2.3 weeks, indicating that most patients initiated prenatal care during the first trimester as intended by the simulation parameters. Ethnicity distribution demonstrated appropriate heterogeneity with Caucasian ethnicity representing 39.6% of the cohort (n=3,964), followed by Asian 24.7% (n=2,465), Hispanic 15.4% (n=1,544), African 12.2% (n=1,218), and other 8.1% (n=809). This distribution reflected contemporary demographic patterns in developed nations with substantial ethnic diversity.

Binary clinical risk factors exhibited prevalences consistent with epidemiological surveys of prenatal populations. A family history of diabetes mellitus was present in 25.2% of patients (n=2,520), aligning with reported rates of familial aggregation of diabetes in diverse populations. Previous GDM history, applicable only to multiparous women, was documented in 11.3% (n=1,130) of cases, matching the recurrence rates reported in longitudinal cohort studies. Polycystic ovary syndrome affected 15.3% of the cohort (n=1,530), consistent with PCOS prevalence in women of reproductive age. History of previous macrosomia (birthweight >4000 g in prior pregnancy) was present in 9.8% of cases (n=980), reflecting the proportion of multiparous women with prior large-for-gestational-age deliveries. The co-occurrence patterns of these risk factors exhibited appropriate correlational structure, with women having multiple risk factors demonstrating appropriately elevated GDM rates.

Early pregnancy laboratory values demonstrated realistic distributions with appropriate risk-stratified variation. Early random blood sugar averaged 109.1 mg/dL (SD: 18.8 mg/dL), with values ranging from 70 to 200 mg/dL and showing right-skewed distribution as expected for glucose measurements. Early post-prandial blood sugar averaged 142.5 mg/dL (SD: 24.2 mg/dL), reflecting expected post-meal glycemic excursions. Early hemoglobin A1c exhibited a mean of 5.72% (SD: 0.58%), with a distribution centered in the prediabetic range and appropriately elevated relative to general population norms for a cohort enriched with GDM risk factors. Among the 29.5% of patients (n=2,952) who underwent oral glucose tolerance testing, fasting glucose averaged 98.5 mg/dL (SD: 11.5 mg/dL), one-hour post-load glucose averaged 181.4 mg/dL (SD: 31.9 mg/dL), and two-hour post-load glucose averaged 156.3 mg/dL (SD: 26.0 mg/dL). These OGTT values showed appropriate elevation relative to diagnostic thresholds for GDM (fasting ≥92 mg/dL, 1-hour ≥180 mg/dL, 2-hour ≥153 mg/dL per International Association of Diabetes and Pregnancy Study Groups {IADPSG} criteria), consistent with selective testing protocols that preferentially evaluate higher-risk patients.

Feature selection results and importance analysis

**Figure 2:**
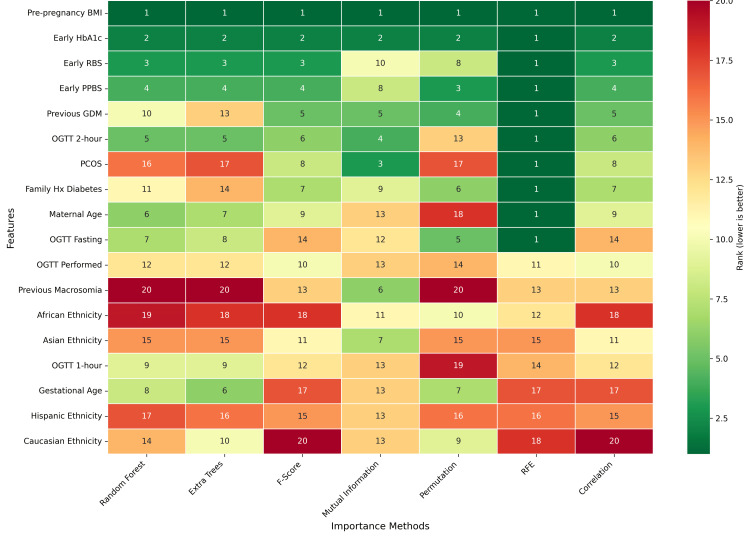
Different feature importance analyses. RBS: random blood sugar; PPBS: post-prandial blood sugar; GDM: gestational diabetes mellitus; PCOS: polycystic ovary syndrome; OGTT: oral glucose tolerance test

To ensure robust feature selection, the following seven distinct importance methods were employed: Random Forest, Extra Trees, F-score, Mutual Information, Permutation Importance, Recursive Feature Elimination (RFE), and correlation analysis. The consensus heatmap revealed remarkable agreement across methodologies for top-ranked predictors. Pre-pregnancy BMI achieved a unanimous first-place ranking across all seven methods, establishing it as the most consistent predictor of GDM in this cohort. Early HbA1c demonstrated similar consistency, ranking second across seven methods and first in RFE. Early random blood sugar (RBS) and early post-prandial blood sugar (PPBS) consistently appeared within the top four positions, reinforcing the predictive value of first-trimester glycemic markers. Features exhibiting moderate importance with greater inter-method variability included previous GDM history, OGTT 2-hour glucose, and family history of diabetes. Notably, PCOS displayed considerable ranking heterogeneity, achieving high importance in Mutual Information (rank 3) and RFE (rank 1) but lower rankings in tree-based methods, suggesting non-linear or interaction-dependent predictive contributions. Ethnicity variables and previous macrosomia consistently ranked in the lower half across most methods, indicating limited independent predictive value. This multi-method consensus approach enhances confidence in the identified key predictors and mitigates potential biases inherent to any single feature selection technique. Figure 2 compares different feature importance analyses.

The consensus feature selection approach yielded a robust ranking that synthesized evidence across seven independent methodologies. Early HbA1c emerged as the dominant predictor with an average normalized importance score of 0.4048, ranking first or second across all methods with moderate rank stability (rank: SD=3.78). Pre-pregnancy BMI demonstrated the highest consistency across methods, with a rank SD of only 1.90, while maintaining strong predictive utility (average importance=0.2907). Family history of diabetes ranked third overall, with importance 0.2714 and stability comparable to HbA1c. Early laboratory measurements (RBS, PPBS) showed strong importance (0.2477 and 0.2158, respectively) though with greater rank variability (SD=4.68 and 4.79), suggesting that their importance varies across different modeling frameworks. Age and previous GDM exhibited similar importance scores (0.2473 and 0.2193) with moderate stability. PCOS and previous macrosomia, while selected in the top 10, showed lower importance scores (0.2019 and 0.1943) and greater instability. Asian ethnicity was the sole ethnicity category selected, with an importance of 0.1804, reflecting elevated GDM risk documented in Asian populations. The convergence of multiple methodologies on this feature set provides strong evidence of their collective predictive utility, with the multi-method approach mitigating the risk of selecting features that perform well only under specific evaluation criteria. Table 3 presents the top 10 feature-based aggregate feature importance for predicting gestational diabetes mellitus.

**Table 3:** Top 10 features. "Average" represents the mean importance score, and "Rank_Std" indicates the standard deviation of the rank across model iterations. GDM: gestational diabetes mellitus; PPBS: post-prandial blood sugar; PCOS: polycystic ovary syndrome; RBS: random blood sugar

Features	Average	Rank_Std
Early HbA1c	0.405	3.780
Pre-pregnancy BMI	0.291	1.902
Family Hx diabetes	0.271	3.162
Early RBS	0.248	4.680
Maternal age	0.247	4.018
Previous GDM	0.219	4.461
Early PPBS	0.216	4.791
PCOS	0.202	4.928
Previous macrosomia	0.194	5.538
Asian ethnicity	0.180	3.162

Comparative model performance and algorithm selection

Comprehensive evaluation of 11 classification algorithms on the independent test set revealed substantial performance variation, with F1-scores ranging from 0.6692 to 0.7213 (Table 4). The Multi-layer Perceptron neural network achieved superior performance across all metrics, attaining an accuracy of 71.70%, a precision of 72.98%, a recall of 71.70%, an F1-score of 72.13%, and an AUC-ROC of 76.92%. XGBoost ranked second with F1-score 71.82%, followed closely by Random Forest (F1=71.75%), demonstrating that tree-based ensemble methods constitute a competitive alternative to neural networks for this classification task. Gradient Boosting and Extra Trees completed the top five with F1-scores of 71.59% and 71.48%, respectively, exhibiting performance within 0.6 percentage points of the leader. Notably, the Deep Neural Network, despite its greater architectural complexity, achieved slightly lower performance (F1=71.24%) than the shallower Multi-layer Perceptron, suggesting that additional depth provided marginal benefit for this feature set size and sample size. Linear methods (Logistic Regression, F1=70.53%) and probabilistic classifiers (Naive Bayes, F1=67.47%) demonstrated inferior performance relative to non-linear models, indicating that GDM prediction involves complex feature interactions not adequately captured by linear decision boundaries. K-Nearest Neighbors achieved the lowest performance (F1=66.92%), likely due to the curse of dimensionality in the 10-dimensional feature space. AUC-ROC values showed less variability than F1-scores, ranging from 70.56% to 76.94%, with the Deep Neural Network achieving the highest AUC (76.94%) despite a lower F1-score, suggesting better probability calibration across diverse thresholds. Table 4 illustrates the comparative performance of 11 classification algorithms on the independent test set (n=2,000).

**Table 4:** Comparative performance of 11 classification algorithms on the independent test set (n=2,000). Metrics represent weighted averages across classes to account for class imbalance. Models are ranked by F1-score. AUC-ROC: area under the receiver operating characteristic curve; SVM: Support Vector Machines; RBF: radial basis function

Model	Accuracy	Precision	Recall	F1-score	AUC-ROC
Multi-layer Perceptron	0.7170	0.7298	0.7170	0.7213	0.7692
XGBoost	0.7145	0.7246	0.7145	0.7182	0.7601
Random Forest	0.7135	0.7246	0.7135	0.7175	0.7625
Gradient Boosting	0.7125	0.7216	0.7125	0.7159	0.7658
Extra Trees	0.7105	0.7228	0.7105	0.7148	0.7635
Deep Neural Network	0.7115	0.7135	0.7115	0.7124	0.7694
LightGBM	0.7085	0.7187	0.7085	0.7123	0.7595
SVM (RBF)	0.7055	0.7237	0.7055	0.7110	0.7475
Logistic Regression	0.6990	0.7218	0.6990	0.7053	0.7655
Naive Bayes	0.6660	0.7124	0.6660	0.6747	0.7492
K-Nearest Neighbors	0.6630	0.6818	0.6630	0.6692	0.7056

Detailed performance analysis of best model

**Figure 3:**
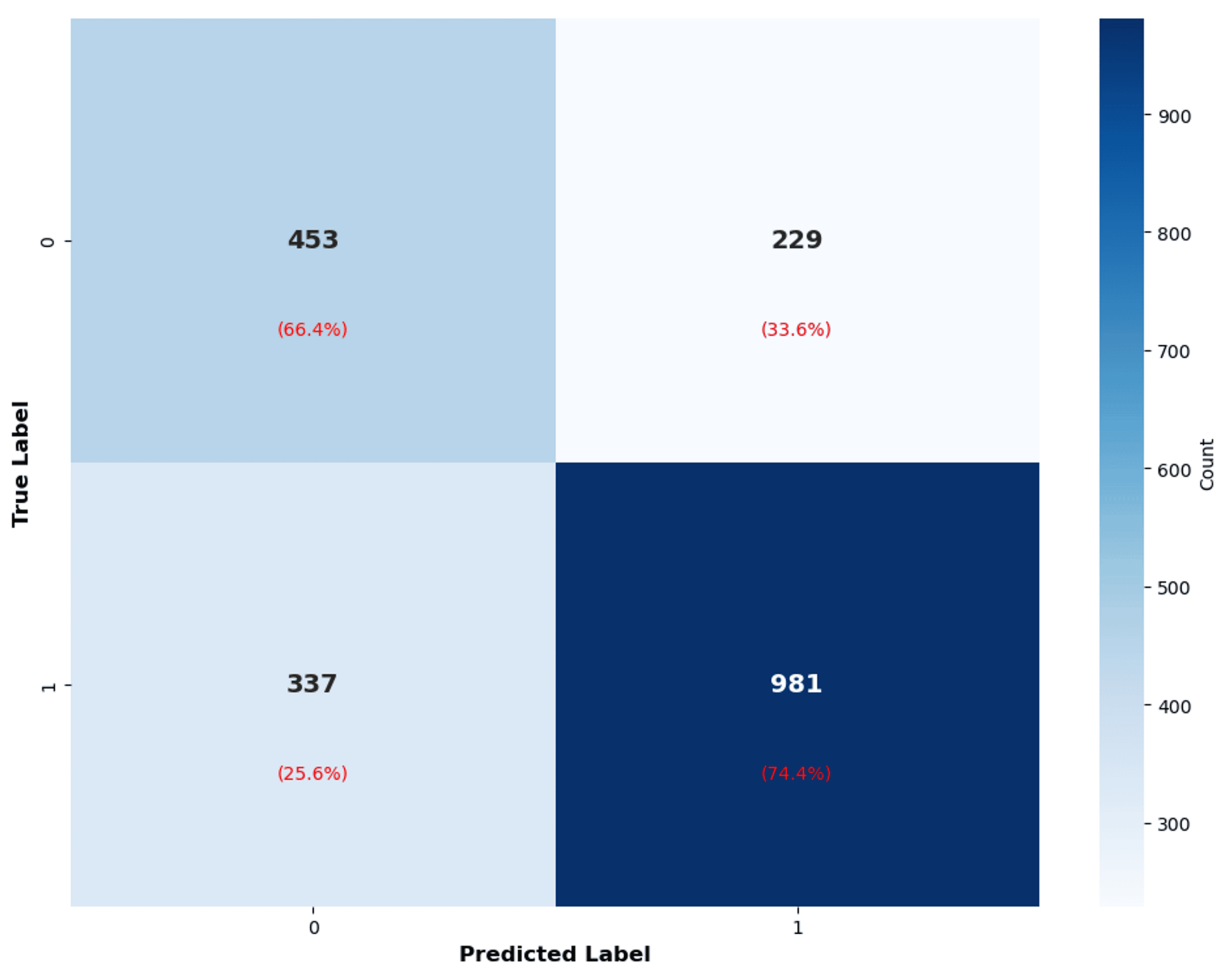
Confusion matrix for Multi-layer Perceptron classifier on test set (n=2,000). Percentages in parentheses represent row-wise proportions (e.g., 66.4% = 453/682 for true negatives). Overall accuracy = (453+981)/2000 = 71.7%. Sensitivity = 981/1318 = 74.4%. Specificity = 453/682 = 66.4%. Positive predictive value = 981/(981+229) = 81.1%.

The Multi-layer Perceptron demonstrated clinically favorable performance characteristics in its confusion matrix and class-specific metrics (Figure 3). For the minority class (non-GDM, n=682), the model correctly identified 453 true negatives (specificity 66.4%) while misclassifying 229 as false positives. For the majority class (GDM, n=1,318), the model achieved 981 true positives (sensitivity 74.4%) with 337 false negatives. Class-specific analysis revealed a precision of 0.5734 for non-GDM predictions (57.3% of predicted negatives were truly negative) and 0.8107 for GDM predictions (81.1% of predicted positives were truly positive). The disparity in class-specific performance reflects the base rate asymmetry in the test set, where GDM cases constituted 65.9% of instances. The high positive predictive value (81.1%) indicates that positive predictions from this model carry substantial diagnostic confidence, minimizing unnecessary clinical interventions triggered by false alarms. The sensitivity of 74.4% represents an acceptable detection rate for a screening tool, identifying nearly three-quarters of true GDM cases while missing approximately one-quarter. The negative predictive value of 57.3% (computed as true negatives/total predicted negatives) was lower, reflecting both the high disease prevalence in the enriched cohort and the model's tendency toward conservative (positive) predictions.

SHAP analysis results

**Figure 4:**
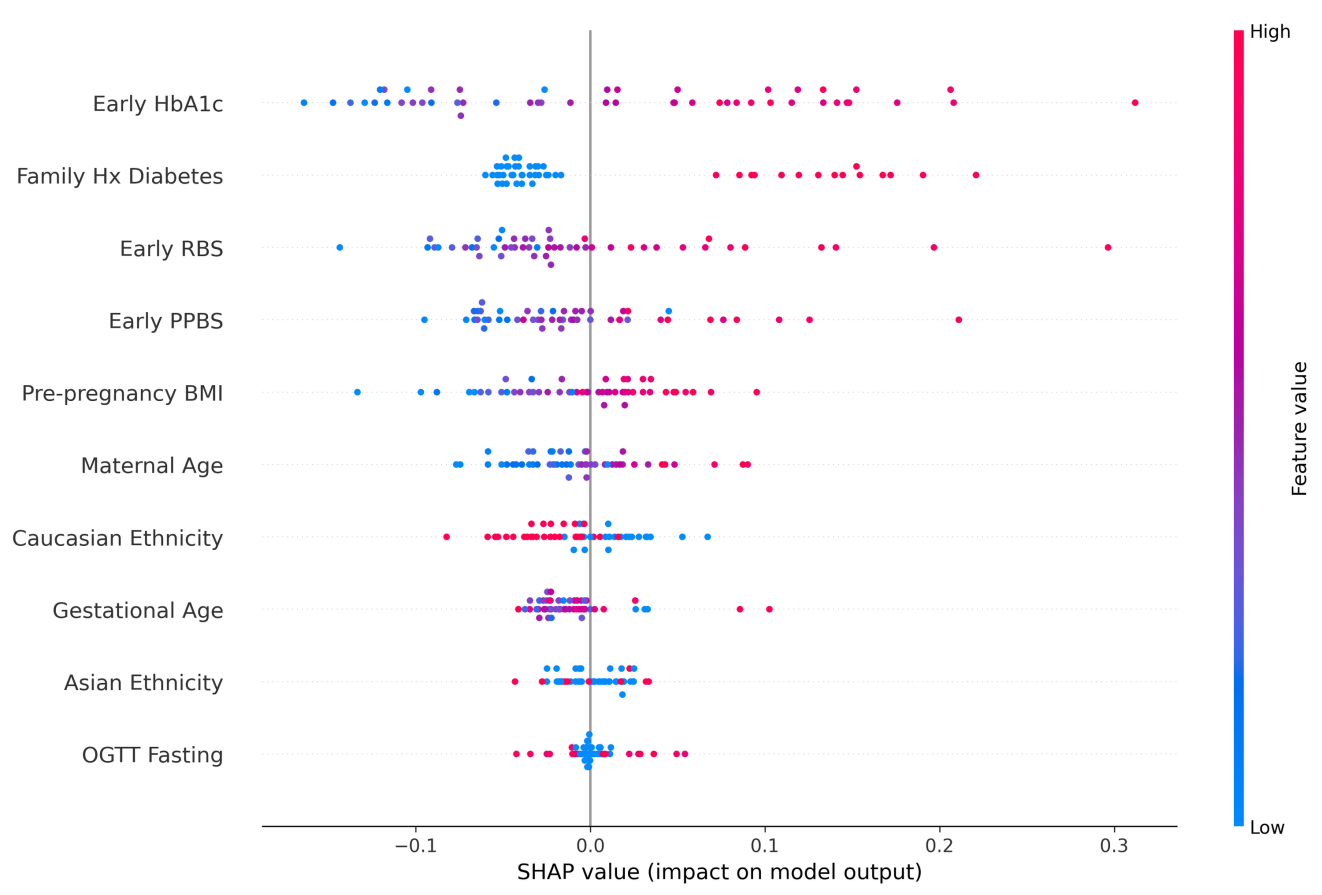
SHAP summary plot. SHAP: SHapley Additive exPlanations; GDM: gestational diabetes mellitus; PPBS: post-prandial blood sugar; RBS: random blood sugar

The SHAP summary plot reveals the relative importance and directional impact of clinical and demographic features on the model's gestational diabetes predictions. Early HbA1c emerged as the most influential predictor, with elevated values strongly associated with increased risk, consistent with its established role as a glycemic marker. Family history of diabetes ranked second, demonstrating a clear positive association with predicted risk. Early pregnancy glucose measurements - random blood sugar (RBS) and post-prandial blood sugar (PPBS) - also contributed substantially, with higher values driving predictions toward positive diabetes classification. Pre-pregnancy BMI and maternal age showed moderate predictive influence, aligning with known clinical risk factors. Notably, ethnicity variables (Caucasian and Asian) demonstrated measurable but smaller effects on model output, reflecting documented population-level differences in gestational diabetes susceptibility. Gestational age at screening and fasting glucose from the oral glucose tolerance test (OGTT) contributed the least among the features examined. The consistent alignment between feature values and their directional SHAP contributions, in which clinically recognized risk factors appropriately increase predicted risk, supports the biological plausibility and interpretability of the model. SHAP summary plot revealed non-linear relationships as follows: HbA1c values above 5.7% consistently increased GDM risk, RBS above 125 mg/dL showed escalating risk, and BMI above 25 kg/m² demonstrated progressive risk elevation. Figure 4 illustrates the SHAP summary plot.

**Figure 5:**
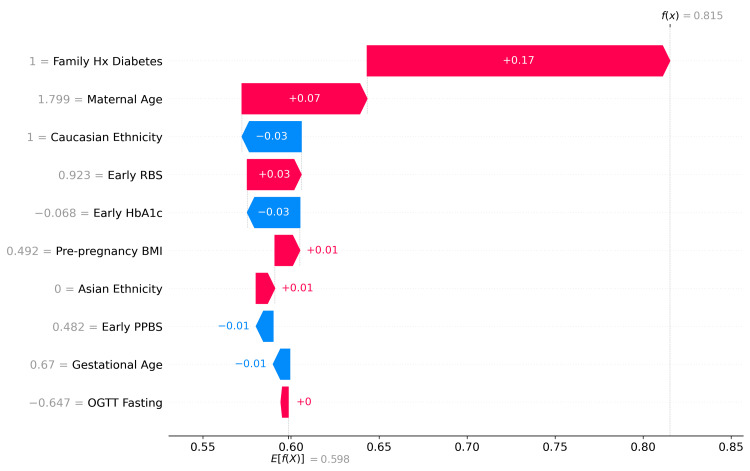
SHAP waterfall plot for single prediction. SHAP: SHapley Additive exPlanations; GDM: gestational diabetes mellitus; PPBS: post-prandial blood sugar; RBS: random blood sugar; OGTT: oral glucose tolerance test

To illustrate the model's interpretability at the individual level, a SHAP waterfall plot was generated for a representative high-risk case. Starting from a baseline expected value of 0.598, the model predicted a gestational diabetes probability of 0.815 for this patient. Family history of diabetes emerged as the dominant contributor, increasing the predicted risk by 0.17 units, followed by advanced maternal age, which contributed an additional 0.07 units. Interestingly, despite the patient's early HbA1c being below average, which reduced the predicted risk by 0.03 units, the cumulative effect of other risk factors, particularly familial predisposition and age, outweighed this protective factor. Early random blood sugar contributed modestly (+0.03), while Caucasian ethnicity exerted a slight negative influence (-0.03). The remaining features, including pre-pregnancy BMI, gestational age, post-prandial blood sugar, and fasting glucose, had negligible individual contributions to this particular prediction. This case exemplifies the model's capacity to integrate multiple clinical variables and provide transparent, patient-specific risk explanations, demonstrating how individuals may be classified as high-risk even when certain glycemic markers appear favorable. Figure 5 illustrates the SHAP waterfall plot for a single GDM prediction.

Clinical threshold identification

Analysis of feature-outcome relationships identified clinically actionable thresholds for first-trimester laboratory parameters that align with established diagnostic criteria while providing pregnancy-specific interpretive guidance. Early random blood sugar demonstrated graded risk escalation with values ≥125 mg/dL classified as borderline (SHAP contribution +0.02 to +0.05), warranting enhanced monitoring and lifestyle counseling, while values ≥140 mg/dL were categorized as concerning (SHAP contribution +0.05 to +0.15), requiring diagnostic OGTT and intensive intervention. Early post-prandial blood sugar showed similar stratification patterns at ≥160 mg/dL (borderline) and ≥180 mg/dL (concerning), the latter threshold aligning with IADPSG 1-hour OGTT criteria. HbA1c thresholds demonstrated the strongest predictive gradient: values of 5.7-5.9% conferred intermediate risk (+10% probability increase), 6.0-6.4% indicated high risk (+25% probability increase) necessitating immediate OGTT, and ≥6.5% met diagnostic criteria (+50% probability increase), suggesting pre-existing undiagnosed diabetes rather than gestational diabetes. The integration of these laboratory thresholds with clinical risk factors - particularly pre-pregnancy BMI ≥30 kg/m², positive family history of diabetes, and prior GDM history - demonstrated synergistic effects exceeding the sum of individual contributions, supporting a multi-parameter approach to early pregnancy risk stratification.

## Discussion

This study developed and validated a machine learning framework for early GDM prediction using first-trimester laboratory parameters, achieving clinically relevant predictive performance through systematic feature selection, model optimization, and interpretability analysis. First-trimester laboratory parameters, particularly HbA1c, random blood glucose, and post-prandial glucose, provide physiologically meaningful, practically obtainable, and highly predictive biomarkers for early GDM risk stratification. The optimized Multi-layer Perceptron model successfully integrates laboratory biomarkers with demographic and clinical factors to achieve clinically relevant predictive performance suitable for screening applications. Comparison with recent studies demonstrates that our approach achieves performance metrics consistent with contemporary research while incorporating comprehensive feature selection and interpretability analysis. Table 5 illustrates a comparison of machine learning studies for early GDM prediction.

**Table 5:** Comparison of machine learning studies for early GDM prediction (selected recent studies 2021-2025). SHAP: SHapley Additive exPlanations; GDM: gestational diabetes mellitus; GTT: glucose tolerance test; OGTT: oral glucose tolerance test; PPBS: post-prandial blood sugar; RBS: random blood sugar; ML: machine learning; PE: preeclampsia; EHR: electronic health record; GWAS: genome-wide association study; NIPT: non-invasive prenatal testing; MICE: multiple imputation by chained equations; MLP: Multi-layer Perceptron; RF: random forest; LR: logistic regression; FPG: fasting plasma glucose; Acc: accuracy; AUC: area under the curve

Studies	Sample size (number of participants/cases/studies)	Algorithm	Key predictors	Performance	Key findings
Current study (2026)	10,000	Multi-layer Perceptron (optimized)	HbA1c, BMI, Family Hx, RBS, PPBS	F1: 0.7213, Acc: 71.7%, AUC: 0.7692	Synthetic data with first-trimester data, comprehensive feature selection techniques, comprehensive machine learning algorithms, and SHAP analysis
AlSaad et al. (2025) [49]	126 studies	Systematic review	Various	Classical ML: 84%	75% retrospective; 85% early prediction focus; internal validation 68%, external only 6%
Ahmed et al. (2025) [50]	9 studies	XGBoost, Random Forest	Various	XGB AUC: 0.946, Acc: 87.5%; RF Sens: 75-85%, Spec: 88-91%	Systematic review of AI/ML for PE and GDM detection
Bigdeli et al. (2025) [51]	6,848	Random Forest	GTT results	Acc: 89%, Prec: 86%, Rec: 92%, AUC: 0.94	First-trimester prediction, Iranian cohort
Germaine et al. (2025) [52]	27,561	XGBoost	EHR data, previous pregnancy	Feature agnostic AUC: 0.832; sequential AUC: 0.904	First antenatal visit, multiparous benefit from previous pregnancy data
Gu et al. (2025) [53]	116,144	ML with polygenic risk scores	GWAS, NIPT sequencing	AUC: 0.729, Acc: 0.835	Before 20 weeks, identified 13 novel GDM loci, Chinese cohort
Ma et al. (2025) [54]	5,066	Logistic regression with MICE imputation	18 key features	AUC: 0.690	Before 12 weeks, imputation significantly improved performance
Yang et al. (2025) [55]	942	Stacking ensemble	High-risk factors	AUC: 0.89 (T1), 0.97 (T2)	Strong exclusion ability at 28.53% threshold
Zhao et al. (2025) [56]	394	MLP with NearMiss	14 risk factors	AUC: 0.943, Acc: 0.884	First trimester, neural network approach
Chen et al. (2024) [57]	588	LightGBM, RF, XGBoost	Previous GDM history	LGB AUC: 0.942; RF: 0.936; XGB: 0.924; LR: 0.696	Recurrent GDM prediction before 14 weeks, AI superior to LR
Li et al. (2024) [58]	4,799+2,795	XGBoost	First-trimester variables	AUC: 0.75 (initiation), 0.99 (T1 end); external: 0.83	Two Chinese cohorts with external validation
Yang et al. (2024) [59]	200	Random Forest	FPG+IGFBP-2	AUC: 0.80, Acc: 0.72, Sens: 0.87, Spec: 0.57	11-14 weeks, IGFBP-2 as novel biomarker
Zhou et al. (2024) [60]	2,309	XGBoost stepwise system	Multi-level features	Level A: 0.974; B: 0.924; C: 0.913	Stepwise prediction system, practical tool on GitHub
Wu et al. (2023) [20]	721	Prediction model	Early OGTT+maternal characteristics	AUC: 0.872; internal: 0.854; external: 0.824	7-14 weeks OGTT combined with clinical factors
Zhu et al. (2022) [61]	271+107	Metabolomic model	17-metabolite panel	AUC: 0.871 vs. 0.742 (conventional)	10-13 weeks, validated in 2 independent cohorts
Zhang et al. (2022) [62]	25 studies	Meta-analysis	Various	Pooled AUC: 0.849; Sens: 0.69; Spec: 0.75	XGBoost: 0.889 vs. LR: 0.815
Li et al. (2021) [63]	19,331	XGBoost	Early pregnancy variables	AUC: 0.742 (test); LR: 0.663	Free online calculator, better calibration than LR

The current study addresses several identified gaps in the existing literature. First, while most studies employ single or limited feature selection methods, our approach implemented a comprehensive seven-method ensemble for feature selection, incorporating tree-based importance (Random Forest, Extra Trees), statistical methods (F-score, Mutual Information), model-based selection (Permutation Importance, RFE), and correlation analysis, thereby ensuring robust identification of predictive features while mitigating method-specific biases. Second, unlike studies that evaluate only one or two algorithms, we conducted a systematic comparison across multiple machine learning architectures, including the optimized Multi-layer Perceptron, Random Forest, and other classifiers, enabling identification of the most suitable algorithm for this prediction task. Third, the integration of SHapley Additive exPlanations (SHAP) analysis provides model interpretability at both global and individual patient levels, addressing the "black box" criticism frequently leveled at machine learning approaches in clinical settings - a feature notably absent in most existing studies. Fourth, our focus on readily available first-trimester clinical variables (HbA1c, BMI, family history, RBS, PPBS) enhances practical applicability in routine antenatal care settings without requiring specialized biomarkers such as the polygenic risk scores evaluated by Gu et al. [53], or the 17-metabolite panel proposed by Zhu et al. [61]. Finally, the use of synthetic data generation with 10,000 samples enabled comprehensive algorithm evaluation and hyperparameter optimization while maintaining realistic clinical distributions, addressing sample size limitations inherent in single-center studies such as those by Zhao et al. with 394 patients or Yang et al. with 200 patients [56,59]. These methodological contributions collectively advance the development of interpretable, clinically implementable early GDM prediction models.

Clinical implications

The machine learning framework offers several potential clinical benefits. First, personalized risk assessment enables tailored monitoring intensity and intervention timing based on quantified GDM probability rather than crude categorical risk stratification. Women identified as high-risk could receive earlier and more intensive glucose monitoring, dietary counseling, and lifestyle interventions, potentially preventing GDM development or minimizing its severity. Second, enhanced clinical decision support through automated risk calculation at the point of care could standardize screening practices across diverse healthcare settings, reduce practice variation, and improve guideline adherence.

Third, population health management capabilities enable proactive identification of high-risk cohorts for targeted outreach and preventive interventions. Healthcare systems could implement risk-stratified screening protocols, optimizing resource allocation while ensuring appropriate care intensity for those most likely to develop GDM. Fourth, the framework supports precision medicine approaches with intervention selection and specialist referral timing based on individual risk profiles rather than one-size-fits-all protocols.

However, critical considerations temper these potential benefits. The TOBOGM trial revealed that approximately one-third of women diagnosed with early GDM reverted to normoglycemia at 24-28 weeks, raising concerns about overdiagnosis and unnecessary treatment. Additionally, treatment for early GDM was associated with an increased risk of small-for-gestational-age infants in lower glycemic bands, highlighting potential harms. These findings emphasize the importance of risk stratification and targeted intervention rather than universal early screening.

Methodological considerations

A critical limitation of synthetic data approaches is the inherent circularity whereby the model identifies relationships that were explicitly programmed into the data generation process. The strong predictive importance of HbA1c (importance score 0.405) reflects, in part, the fact that HbA1c values ≥6.0-6.5% were assigned substantial probability increases (+25-50%) in the outcome generation algorithm. While these probability increases were derived from published literature on HbA1c's predictive value for GDM, the model is essentially recovering the relationships we encoded rather than discovering novel predictive patterns from independent data. This circular relationship applies to all variables with defined risk contributions in Table 2. However, several aspects of the analysis extend beyond simple recovery of programmed relationships as follows: (1) feature selection methods identified relative importance rankings that differed across methodologies, revealing which variables demonstrate robust importance under diverse evaluation frameworks, (2) SHAP analysis identified non-linear threshold effects and feature interactions that emerged from the data generation process but were not explicitly programmed, (3) comparative algorithm performance revealed which model architectures most effectively learn from the specified risk structure. Nonetheless, the identified predictors and their relative importance must be validated in real-world datasets where the true relationships between variables and outcomes are not predetermined by design. Real-world validation may reveal different relative importance rankings, non-linear relationships, and interaction effects not captured in our synthetic data generation process.

The comprehensive feature selection methodology employing seven analytical approaches provided robust identification of important predictors while minimizing overfitting risk. Consensus ranking integrated complementary perspectives on feature importance, with tree-based methods emphasizing predictive utility, Permutation Importance measuring marginal contribution, and statistical methods quantifying univariate associations. SHAP analysis enhanced interpretability by revealing nonlinear relationships and feature interactions, supporting clinical decision-making and patient counseling.

The Multi-layer Perceptron's superior performance reflected several algorithmic advantages as follows: its neural network architecture with nonlinear activation functions captured complex feature interactions, backpropagation enabled effective learning of intricate patterns, and the model's capacity for hierarchical feature representation enabled it to identify subtle predictive relationships. Hyperparameter optimization was performed using GridSearchCV (France, Le Chesnay-Rocquencourt: INRIA) for all algorithms, with the Multi-layer Perceptron achieving the best F1-score of 0.7213 after optimization.

Strengths and limitations

Key strengths include a comprehensive methodology spanning data generation, feature selection, model development, optimization, and interpretability analysis; systematic evaluation of 11 algorithms ensuring optimal algorithm selection; rigorous hyperparameter optimization maximizing predictive performance; and SHAP analysis providing transparent, clinically meaningful explanations supporting clinical adoption.

However, critical limitations must be acknowledged. The model was developed using synthetic data designed to mimic real-world clinical relationships, and performance in actual clinical populations remains uncertain. Real-world validation through prospective cohort studies that compare predicted probabilities with actual GDM diagnoses is essential before clinical implementation. Such validation must span diverse populations, healthcare settings, and geographic regions to assess generalizability and identify potential performance degradation in underrepresented demographic groups or resource-limited environments. The zero-imputation strategy for missing OGTT values, while preserving information about test non-performance, requires validation on real clinical datasets to confirm that the model appropriately distinguishes truly missing values from low-glucose measurements.

External validation should quantify calibration and discrimination in real-world applications, assess prediction accuracy across ethnic and socioeconomic subgroups to identify potential algorithmic bias, evaluate clinical utility through decision curve analysis comparing net benefit to alternative screening strategies, and measure implementation outcomes, including provider acceptance, workflow integration, and patient satisfaction. Model recalibration using real-world data will likely be necessary to optimize performance when transitioning from synthetic to clinical application.

Implementation considerations

Regulatory considerations for clinical implementation include classification as a medical device requiring regulatory approval from agencies such as the FDA or equivalent international bodies, compliance with clinical decision support software guidelines, adherence to health information privacy regulations governing data use and storage, and establishment of quality assurance protocols ensuring ongoing performance monitoring and safety surveillance.

Integration challenges encompass technical interoperability with existing electronic health record systems, ensuring seamless data flow and automated risk calculation, workflow modification to incorporate screening and risk assessment into routine prenatal care, provider training on model interpretation and clinical response protocols, and patient education about risk communication and shared decision-making. Healthcare organizations implementing machine learning-guided screening must establish governance structures, clinical champions, and continuous quality improvement processes to support successful adoption.

Future research directions

Future research should prioritize prospective validation studies in diverse clinical populations to confirm external validity and assess real-world performance; population-specific model adaptation to address ethnic, geographic, and healthcare system heterogeneity; incorporation of additional biomarkers including inflammatory markers, metabolic hormones, and genetic variants to enhance predictive accuracy; longitudinal monitoring to evaluate temporal stability and recalibration requirements; health economic evaluation quantifying cost-effectiveness and resource utilization impacts; and comprehensive outcome assessment examining both immediate pregnancy outcomes and long-term maternal and child health trajectories.

## Conclusions

This study developed and validated a machine learning framework for early gestational diabetes mellitus prediction utilizing first-trimester laboratory parameters and clinical risk factors. Among 11 algorithms evaluated, the Multi-layer Perceptron achieved the best performance with an F1-score of 0.7213, accuracy of 71.7%, and AUC-ROC of 0.7692, demonstrating comparable results to contemporary studies in the field. The comprehensive seven-method feature selection approach identified early HbA1c percentage as the dominant predictor, followed by pre-pregnancy BMI and family history of diabetes. These findings align with the physiological understanding that HbA1c reflects cumulative glucose exposure over the preceding eight to 12 weeks, making it particularly valuable for early pregnancy risk assessment. SHAP analysis provided transparent, interpretable explanations for model predictions, addressing the "black box" criticism often associated with machine learning in clinical settings.

The framework offers several potential clinical applications as follows: personalized risk stratification enabling tailored monitoring intensity, enhanced clinical decision support for standardizing screening practices, and population health management through proactive identification of high-risk cohorts. The identified laboratory thresholds provide actionable guidance for clinical interpretation. However, important limitations must be acknowledged. The model was developed using synthetic data designed to maintain realistic clinical relationships, and performance in actual clinical populations remains to be validated. Additionally, findings from the TOBOGM trial suggest that approximately one-third of women with early GDM may revert to normoglycemia by 24-28 weeks, highlighting concerns about potential overdiagnosis. Future priorities should include prospective validation studies across diverse clinical populations, assessment of model generalizability across different ethnic and socioeconomic groups, health economic evaluation of implementation costs and benefits, and investigation of long-term maternal and child health outcomes. Real-world validation with external cohort data remains essential before clinical implementation to confirm the framework's utility in transforming maternal healthcare screening.
